# Ocular bio-lubricating materials: from lubrication mechanism to dry eye syndrome treatment

**DOI:** 10.1093/rb/rbaf121

**Published:** 2025-11-24

**Authors:** Yanxin Zhang, Suoqi Ding, Xinyi Wang, Jin Li, Hao Yu, Weifeng Lin

**Affiliations:** State Key Laboratory of Bioinspired Interfacial Materials Science, Bioinspired Science Innovation Center, Hangzhou International Innovation Institute, Beihang University, Hangzhou 311115, China; State Key Laboratory of Bioinspired Interfacial Materials Science, School of Chemistry, Beihang University, Beijing 100191, China; State Key Laboratory of Bioinspired Interfacial Materials Science, School of Chemistry, Beihang University, Beijing 100191, China; State Key Laboratory of Bioinspired Interfacial Materials Science, Bioinspired Science Innovation Center, Hangzhou International Innovation Institute, Beihang University, Hangzhou 311115, China; State Key Laboratory of Bioinspired Interfacial Materials Science, School of Chemistry, Beihang University, Beijing 100191, China; School of Instrumentation and Optoelectronic Engineering, Beihang University, Beijing 100191, China; Imeik Technology Development Co., Ltd., Beijing 102299, China; State Key Laboratory of Bioinspired Interfacial Materials Science, Bioinspired Science Innovation Center, Hangzhou International Innovation Institute, Beihang University, Hangzhou 311115, China; State Key Laboratory of Bioinspired Interfacial Materials Science, School of Chemistry, Beihang University, Beijing 100191, China

**Keywords:** bio-lubricants, lipids, hydration lubrication, dry eye syndrome, ocular lubrication

## Abstract

The human eye, a mechanically dynamic and physiologically vital organ, sustains continuous mechanical activity through repetitive blinking—averaging approximately 20 000 cycles daily, while exhibiting exceptional lubrication performance characterized by an ultralow coefficient of friction (<0.01). This remarkable lubricating functionality is mediated by the tear film, a multifunctional biological lubricant combining boundary lubrication mechanisms (via adsorbed mucins and lipids) and fluid film lubrication mechanisms to minimize friction and wear, and preserve ocular surface integrity. Failure of such ocular lubrication can cause tear film instability or ocular surface damage, leading to discomfort, visual dysfunction and dry eye syndrome. Ocular lubrication involves multiple structures and lubricants with highly complex biomolecular interactions. Insights into the structure of eyes, lubricant composition and causes of functional impairments are essential for addressing friction-related diseases in biological systems. This review examines ocular lubrication by first exploring the biological structure of the eyes and typical lubrication modes. Then, the characterization tools, such as tribometer, atomic force microscope and surface force balance in the field of ocular lubrication, are introduced, followed by a comparison of their working principles, applicable conditions and application fields. Finally, the specific causes of dry eye syndrome are outlined, along with current bio-lubricants, contact lenses and other ocular-inspired bio-lubricating materials.

## Graphical abstract

**Figure:**
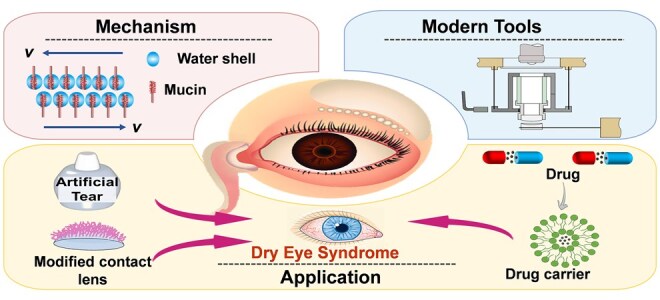

## Introduction

Friction is a universal phenomenon in living and non-living systems. While occasionally advantageous, it often results in energy loss and wear, presenting challenges in industrial engineering [1, 2]. Lubrication science addresses these issues by systemically developing advanced lubricants and surface engineering strategies to minimize friction losses and ensure the reliability of mechanical systems under operational conditions. Biological tribology, a distinct field from traditional tribology, focuses on human anatomy and health. Conceptualized by Dawson in 1973, biotribology has evolved into a cornerstone of biomedical research, with human biotribology constituting a pivotal subdomain critical for health diagnostics and physiological monitoring. Friction critically influences the functionality of diverse biological interfaces, including the ocular surface, oral mucosa, esophageal lining, intestinal epithelium, vascular endothelia, musculoskeletal tissues (tendons, muscles, articular cartilage) and synovial joints. In healthy individuals, biological lubricants ubiquitously distribute across these interfaces and strategically mitigate shear-induced tissue damage through molecular mechanisms tailored to their microenvironment [3]. Ocular lubrication exemplifies a sophisticated subsystem within this paradigm, where the ultralow coefficient of friction (COF < 0.01) [4] is maintained during eyelid-globe kinematics despite demanding operational parameters: sustained low contact pressures (∼0.3–7 kPa), high blinking frequencies (15–20 cycles/min) and elevated shear rates (1–150 mm/s) [5, 6]. Inadequate or dysfunctional ocular lubrication can cause dryness, burning and corneal vasodilatation, collectively manifesting as dry eye syndrome (DES), a chronic ocular surface disorder with profound impacts on visual function and quality of life.

DES now represents a substantial global burden, with epidemiological studies reporting prevalence rates as high as 50% across diverse populations. While historically linked to aging, its rising incidence among younger demographics has elevated DES to a critical public health priority [7]. This multifactorial disorder manifests through compromised ocular lubrication and tear film dysfunction, including insufficient tear production, excessive evaporation and abnormal composition, which disrupts the ocular surface microenvironment and causes tissue damage. Therapeutic strategies prioritize restoration of tear film homeostasis to heal the epithelium, normalize the ocular surface, prevent complications like permanent epitheliopathy and alleviate irritation symptoms such as foreign body sensation and redness. Common DES treatments include tear replenishment, anti-inflammatory therapies, specialized soft contact lenses (SCLs), surgical interventions and lifestyle modifications [8]. Among these, tear replenishment using artificial tears is the most convenient and effective way to relieve DES. Artificial tears, a class of ophthalmic lubricant formulations to mitigate symptoms of DES and maintain ocular surface hydration, predominantly rely on viscosity-enhancing polymers. Key compositional agents include methylcellulose, hyaluronic acid (HA), polyacrylic acid derivatives, dextran, trehalose, hydroxypropyl-guar (HP-guar) and hydroxypropyl methylcellulose (HPMC), which collectively improve tear film stability and moisture retention [8, 9]. However, frequent use of artificial tears may cause unwanted side effects, emphasizing the need for developing long-lasting bio-lubricating materials [10].

From a lubrication mechanism perspective, ocular surface lubrication relies on the ability to reduce the friction and shear forces of the tear film during blinking. Abnormal tear composition disrupts the lubrication mechanism, leading to ocular surface damage. Therefore, understanding its nanoscale lubrication behavior is crucial for developing efficient ocular lubricants. Precise measurement techniques are essential for evaluating ocular surface lubrication performance and understanding the microscopic manifestations of lubrication mechanisms. Commonly used instruments include universal mechanical tester (UMT), atomic force microscopy (AFM) and surface force balance (SFB). These tools can measure friction coefficients at both the macroscopic and the nanoscale levels, characterize viscoelastic properties and analyze the lubrication behavior of extremely thin tear films. They not only deepen our understanding of the pathophysiological mechanisms of DES but also provide critical evidence for the development of more effective artificial tears and bio-lubricating materials.

This paper reviews studies over the past 5–10 years. Firstly, ocular lubrication mechanisms and synergistic effects of multiple mechanisms are discussed. Secondly, commonly used technical tools (tribometer, AFM and SFB) are reviewed for ocular lubrication. In the third section, we systematically describe the ocular anatomy and pathophysiological mechanisms underlying DES while conducting a comprehensive investigation into the molecular mechanisms of bio-lubricants in maintaining ocular surface homeostasis. In addition to elucidating the fundamental biophysical principles governing ocular lubrication, this review critically evaluates emerging therapeutic applications, particularly their innovative integration with advanced ocular drug delivery platforms and biomimetic nanomaterials engineered to replicate natural lubrication processes.

## Lubrication of the ocular surface and DES

Ocular lubrication is an essential component of the biological lubrication system. During blinking, the ocular surface primarily functions through the lubricating action of the tear film. At this time, the contact surfaces are completely isolated by a viscous fluid layer, and the fluid film pressure is in equilibrium with external loads. Developing microscopic lubrication models aids in understanding ocular lubrication mechanisms and offers insights into treating DES caused by lubrication deficiencies. Ocular lubrication models can be categorized into boundary lubrication, mixed lubrication, and fluid lubrication according to Stribeck curves [11]. In the boundary lubrication stage, bio-lubricating molecules in the boundary layer primarily govern the process. The lubricating film is thinner than the surface roughness of the contact pair, resulting in full surface contact and a high COF. In the mixed lubrication stage, a certain amount of lubricating medium exists between the contact surfaces, but some surface asperities remain in contact. The lubricant entirely separates the two surfaces when the film thickness exceeds the surface roughness. At this stage, the COF is low. Under constant load, the COF increases slightly with rotational speed [12]. This section first introduces the composition and structure of the ocular surface, then provides detailed explanations of fluid, boundary lubrication and hydration lubrication mechanisms, and finally introduces the causes and classification of DES.

### Ocular surface structure

**Figure 1.**
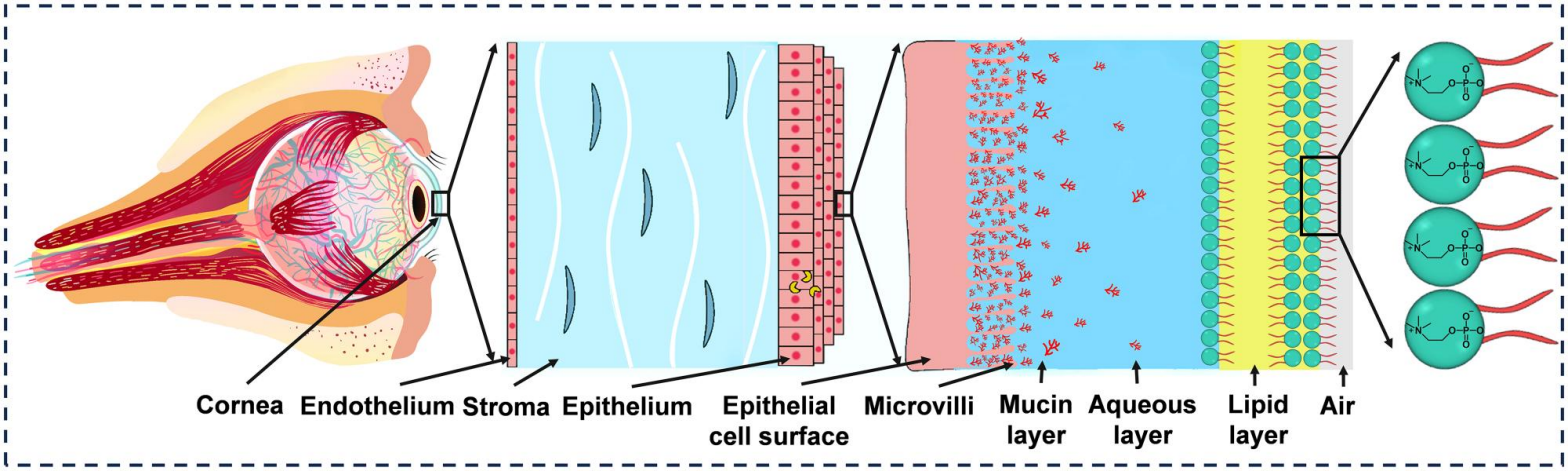
Schematic diagram of the ocular tear film structure. The ocular tear film has a typical three-layered structure, with the epithelium, the intermediate aqueous layer, and the outer lipid layer in order from the inside to the outside. Reproduced with permission [14]. Copyright 2020, Elsevier.

The proper functioning of the ocular lubrication system relies on minimizing friction and wear between the cornea and eyelid and maintaining effective tear film lubrication. Consequently, the corneal and tear film structures are key subjects in ocular lubrication studies. The surface of the eyes comprises the cornea and conjunctiva, featuring a multilayered hierarchical structure with a moist epithelium on its anterior surface (Figure 1) [13].

The corneal epithelium, measuring approximately 50–60 μm in thickness, features a uniform, smooth and regular surface [15]. Being exposed tissue, it is vulnerable to damage from direct environmental contact. The epithelium and the tear film exhibit a symbiotic relationship, with the tear film acting as both a protective barrier and a hydration source for the ocular surface [16, 17]. The classical three-layer model of the ocular tear film is well established [14], consisting of an epithelial layer, an intermediate aqueous layer, and an outer lipid layer. The polar components of the mucin and phospholipid layers adhere to the epithelial surface and orient toward the intermediate aqueous layer. Mucins, a diverse family of proteoglycans, are characterized by a protein backbone densely decorated with O-linked oligosaccharides forming a characteristic bottlebrush (BB) structure [18]. These molecules bind to epithelial cells, where their densely packed, highly hydrated polysaccharide chains covalently form a hydrophilic matrix. This structure provides elasticity and osmotic resistance to compression while creating a smooth, shear-resistant interface. The lipid layer is primarily composed of phosphatidylcholine (PC). Each PC molecule features two hydrophobic acyl tails and a hydrophilic headgroup containing a negatively charged phosphate group and a positively charged choline moiety. The outer lipid layer prevents water evaporation and fluid spillage in the aqueous layer [19]. The tear film significantly reduces friction between the eyelid and the cornea. However, abnormalities in the tear film layers often lead to symptoms of inadequate lubrication, reflecting instability and deficiencies in essential components [20].

### Fluid film lubrication

**Figure 2.**
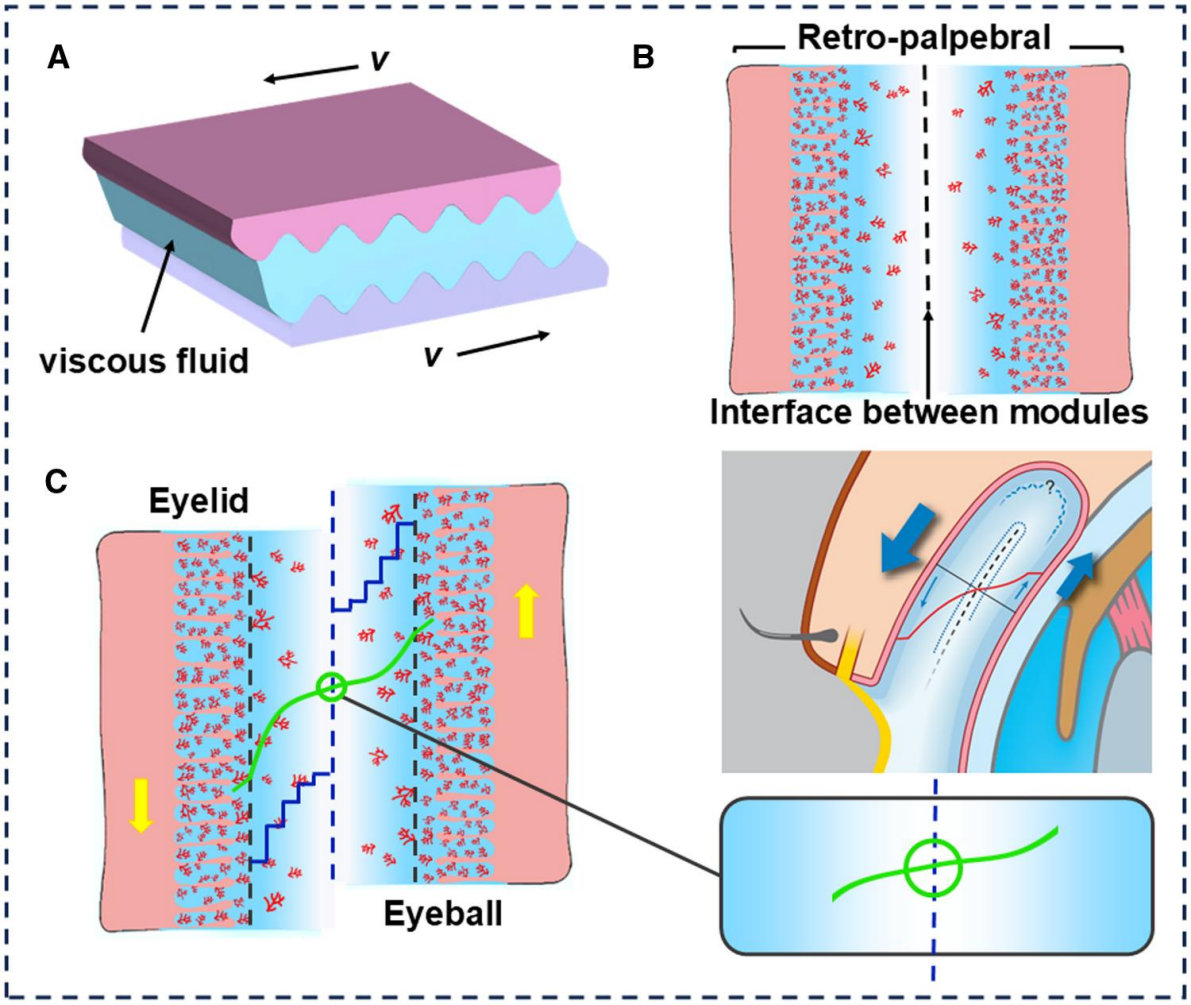
The classical model of fluid film lubrication and the fluid film lubrication model of the eye. (**A**) In the classical fluid film lubrication framework, interfacial shear dynamics are governed by sliding velocity (*v*). (**B**) Sagittal cross-sectional analysis of the retro-palpebral recess reveals a bilaminar architecture exhibiting bilateral mirror-image symmetry. (**C**) The sagittal section of the muco-aqueous in retro-palpebral recess shows the interface between the modules with superimposed velocity profiles (curved blue), which gradually slow down in response to internal friction in the dacron. At the slipping interface, respective fluids may move at differing and contrary velocities. (**B**) and (**C**) Reproduced with permission [22]. Copyright 2008, Elsevier.

Fluid film lubrication, also known as hydrodynamic lubrication, is considered one of the most effective methods for minimizing friction and reducing surface wear in mechanical systems (Figure 2A). This principle finds a remarkable parallel in biological systems, particularly in ocular lubrication. The effectiveness of fluid film lubrication primarily depends on the formation of a stable tear film with adequate thickness between the eyelid and the surface of the eyeball, which usually occurs during blinks or eye movements with high relative velocities (as illustrated in Figure 2B and C). This dynamic process generates hydrodynamic pressure within the fluid layer, which serves load support, surface separation, and tribological optimization [21].

This movement transfers kinetic energy to a low-viscosity intermediate region on the surface of the eyeball, forming a spherical fluid film concentric with the surface of the eyeball [22]. The shear-dependent rheological properties of the fluid film demonstrate velocity-dependent viscoelastic behavior, where the interfacial shear strength exhibits a positive correlation with relative sliding velocity. They can be written as $\tau =K{\left(\frac{du}{dy}\right)}^{n}$, where τ is the shear stress, K is the consistency factor, $\frac{du}{dy}$ is the shear rate, and the flow index *n* is approximately 0.6–0.9 (exact values vary with individual and tear film composition).

The hydrodynamically lubricated sliding interface, characterized by slow relative motion between the fluid films, transforms the high-speed flow induced by blinking into a low-speed peristaltic motion. Residual shear effects on the epithelium occur only between slow-flowing mid-region fluids, not between fast-moving solid epithelia in direct contact. However, the complex morphology and low elasticity of biological friction surfaces render a fluid-only perspective insufficient for analyzing the frictional processes of the system, necessitating further refinement of the ocular lubrication model.

### Boundary lubrication

Fluid film lubrication mechanisms operate through hydrodynamic separation of friction interfaces, whereas boundary lubrication is governed by molecular boundary layers at contacting surfaces. In boundary lubrication regimes, interfacial interactions depend on an ultra-thin boundary film (1–100 nm) that becomes functionally dominant when fluid film lubrication fails under high loads or low sliding velocities. Under such conditions, the bulk fluid properties of the lubricant become negligible, with surface asperities and boundary film characteristics dictating frictional behavior. The synergistic effects of surface topography and boundary film architecture, independent of lubricant viscosity or density, determine the COF of this regime.

**Figure 3.**
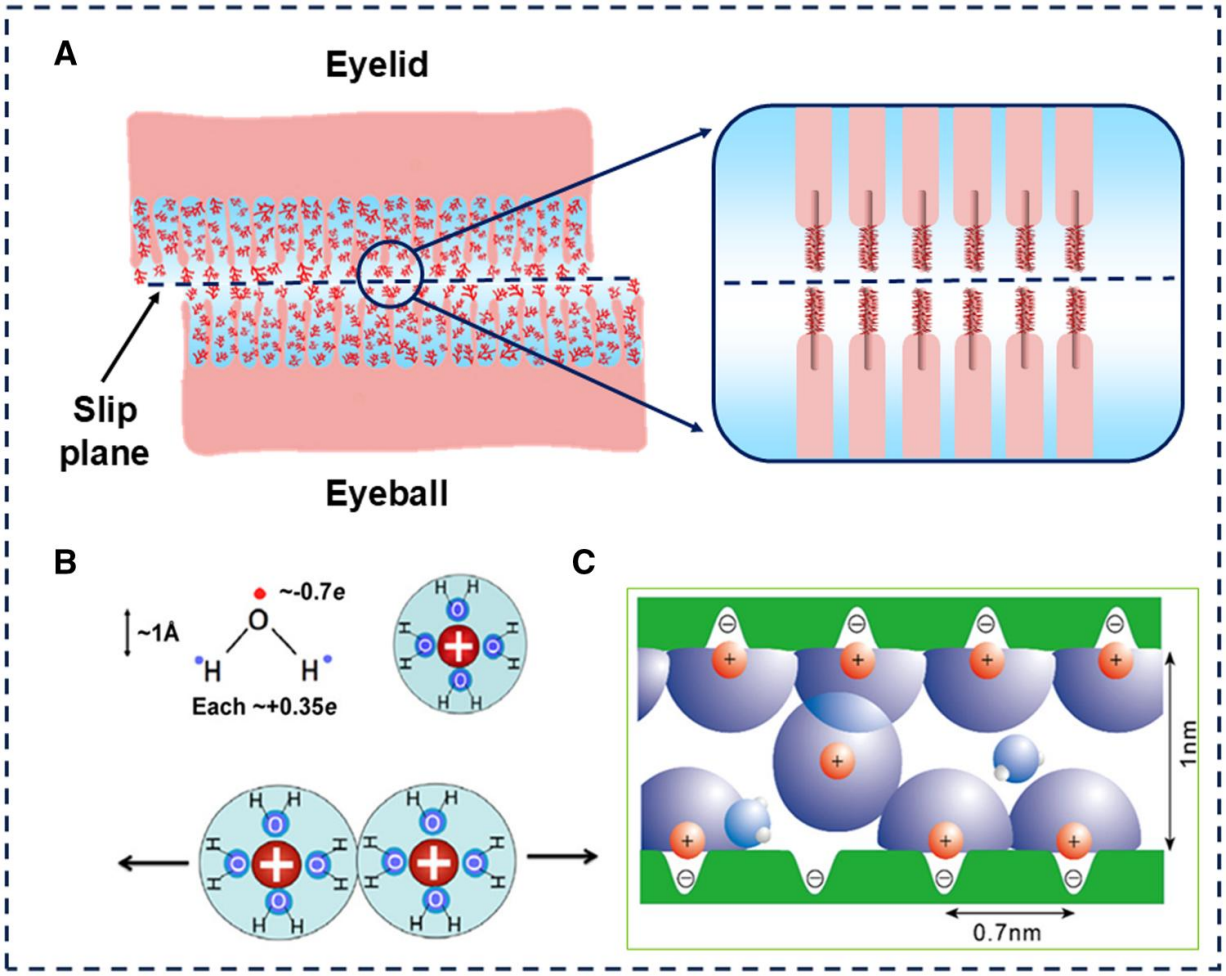
The classical mode of the ocular boundary lubrication and hydration lubrication model. (**A**) In the ocular boundary lubrication mechanism, in direct contact with the sliding plane is the mucin layer, which is tightly bound to the epithelium and ensures a lubricated state of the eyes. (**B**) Illustrating the large dipole moment of a water molecule due to the difference in the electronegativity of atoms. (**C**) A hydration shell of water molecules surrounding a positively charged ion. (**B**) and (**C**) Reproduced with permission [24]. Copyright 2022, American Chemical Society.

Boundary film formation involves three principal mechanisms: physical adsorption (physisorption), chemical adsorption (chemisorption) and tribochemical reactions [4]. The mucin layer of the tear film exhibits a finely layered, multi-scale mucin assembly structure, with mechanisms such as the excellent adsorption, hydration capacity, and viscoelasticity of the mucin network, contributing to lubrication at the ocular interface. Mucoproteins on the surface of corneal epithelial cells form a structurally ordered tear film, which evenly distributes tears across the cornea and conjunctiva with each blink, protecting them from damage caused by blinking. These supramolecular assemblies exhibit a characteristic architecture: a central anionic hydrophilic mucin core flanked by amphiphilic molecules, collectively generating repulsive forces (as illustrated in Figure 3A). Electrostatic repulsion between negatively charged sialic acid residues and steric hindrance from glycosylated mucin domains synergistically mitigate intermolecular adhesion during blink cycles [23].

Additionally, the amino and carboxyl termini scattered throughout the mucin molecules exhibit significant hydrophobic properties. This amphiphilic block copolymer structure, composed of hydrophilic glycosylated regions and hydrophobic non-glycosylated regions, enables strong adhesion to various substrate surfaces through multiple intermolecular forces, including hydrogen bonding, hydrophobic interactions, and electrostatic attraction. Liu *et al.* [25] developed an *in vitro* mucin-deficient simulated ocular surface model and investigated the impact of mucin deficiency in ocular lubricants on ocular surface properties. The study establishes that mucin dysfunction in DES primarily disrupts nanoscale tribological homeostasis rather than macroscopic wettability.

### Hydration lubrication

While the classical boundary lubrication mechanism is insufficient to rationalize the ultralow COF observed in the ocular system, the emergence of hydration lubrication theory, which posits that interfacial water layers dynamically reorganize under shear to minimize energy dissipation, offers a robust mechanistic framework for this phenomenon [26, 27]. Hydration lubrication mediated by hydrated boundary species (such as ions, lipids, and amphoteric polymers with charged or amphoteric groups) significantly reduces friction in aqueous environments and enables interfacial super-lubrication [28]. The angular shape of water molecules and the significant electronegativity difference between hydrogen and oxygen make water a dipole molecule (as shown in Figure 3B). Through dipole–charge interactions, water molecules form transient bonds with charged bodies in solution, creating short-range ordered sparse layers known as hydration layers or shells. Although the hydrated layer exchanges rapidly with nearby free water molecules (as shown in Figure 3C), it remains tightly bound to the charge, making it difficult to disrupt. This occurs because the hydrated layer significantly reduces the self-energy (or Born energy) of confined charge, requiring substantial energy (dehydration energy) to remove it [24]. When two hydrated surfaces are compressed, the rigid hydrated layer resists high normal stresses (as shown in Figure 3C), far exceeding those in most biological systems. At the same time, they retain their fluidity during shear [26, 29, 30]. This shearing of hydration shells rather than direct solid–solid contact reduces interfacial shear stresses by 2–3 orders of magnitude compared to dry friction regimes.

Hydration lubrication plays an important role in facilitating low-friction between the corneal surface and the brush-like zone of the eyelid. In ocular hydration lubrication, the mucus layer of the tear film features a multi-scale mucin assembly that provides both lubrication and protection. Specifically, mucins in the mucus layer adsorb onto the corneal epithelial surface through hydrophobic interactions at their globular termini, while their hydrophilic glycosylated regions extend outward to create a dynamic brush-like structure [31]. The hydrophilic domains tightly coordinate with water molecules, forming a tough hydration shell that resists high compressive load and remains fluid under eye movements. At the same time, the amphoteric nature of the components of the lipid head groups can also ensure high water-retention capacity through a hydration lubrication mechanism, which synergizes with the mucin layer to exert lubricating properties in the eye. Indeed, hydration lubrication is essential in living organisms, providing critical lubrication for numerous biological activities. Physiological activities such as joint movement, oral wetting, and gastrointestinal peristalsis rely on hydration lubrication facilitated by bio-lubricating molecules in tissue fluids. Hydration lubrication is also extensively utilized in functional polymeric materials, including stimulus-responsive lubricants, biomedical lubricants and biomimetic lubricants, such as heat- and pH-sensitive microgels [32], biomedical lubricant coatings [33] and biomimetic articular joint [34].

### Causes and classification of DES

DES is caused by the complex interaction of endogenous and exogenous factors, with hundreds of potential etiological factors. Among these, dysfunction of the ocular surface lubrication function is the primary etiological factor, presenting significant challenges for treatment. Specifically, ocular tear film instability is a central pathogenesis of ocular surface damage, leading to symptoms such as lubrication failure and visual impairment [9, 35]. Sustaining optimal lubrication is essential to maintain the homeostasis of biological surfaces under continuous external stress.

DES can be classified into two main types based on the cause of tear film dysfunction. The first type is aqueous deficiency dry eye, which can be further classified into two subtypes: Sjögren’s syndrome-related (SSDE) and non-Sjögren’s syndrome-related (NSSDE). SSDE is a systemic autoimmune disease characterized by T-lymphocyte infiltration of the lacrimal and salivary glands, leading to cell apoptosis and secretory dysfunction; NSSDE includes primary or secondary lacrimal gland insufficiency, lacrimal duct obstruction and reduced reflex secretion, commonly seen in various etiologies such as age-related degeneration, surgical injury, neuropathy or conjunctival scarring [36].

The second type is evaporative dry eye, characterized by excessive tear evaporation and instability due to lipid layer dysfunction [9, 37]. These two types of dry eye may overlap, increasing tear film osmolarity and predisposing to other ocular disorders, such as inflammation, tear film instability, redness and visual impairment [20, 38]. Damage to the ocular surface epithelium, leading to mucin loss, can result in mucin-deficient DES [39]. The most common form of DES combines two or more factors. Besides tear film dysfunction from reduced content and composition, contact lens wear can cause or exacerbate dry eye symptoms [40].

Despite their widespread use, prolonged contact lens wear can cause discomfort, DES, allergic conjunctivitis, and other ocular disorders [41]. Clinical studies reveal that DES patients exhibit a significantly thinner ocular lipid layer compared to healthy controls, predisposing them to discomfort during lens wear due to impaired tear film continuity over the lens surface [42]. Contact lenses with a thickness approximately 10-fold greater than the natural tear film partition the tear film into anterior and posterior layers. This structural disruption destabilizes tear film dynamics, exacerbating ocular surface desiccation and friction [40, 43]. Two critical biomechanical challenges arise. First, mucin deficiency within the anterior layer compromises lens surface wettability, impairing lubrication efficacy and amplifying interfacial friction between the eyelid and ocular surface [44]. Second, reduced tear volume posterior to the lens leads to precarious thinning of the tear film, elevating shear stress at the lens-cornea/conjunctiva interface [43, 45].

Due to its complex etiology, curing dry eye entirely is challenging. The goal of treating dry eye is to alleviate symptoms in mild cases and preserve visual function in severe cases. By removing the cause and treating the underlying disease, DES can be alleviated with artificial tears, therapeutic contact lenses, and targeted medication.

## Modern tools for ocular tribology

**Figure 4.**
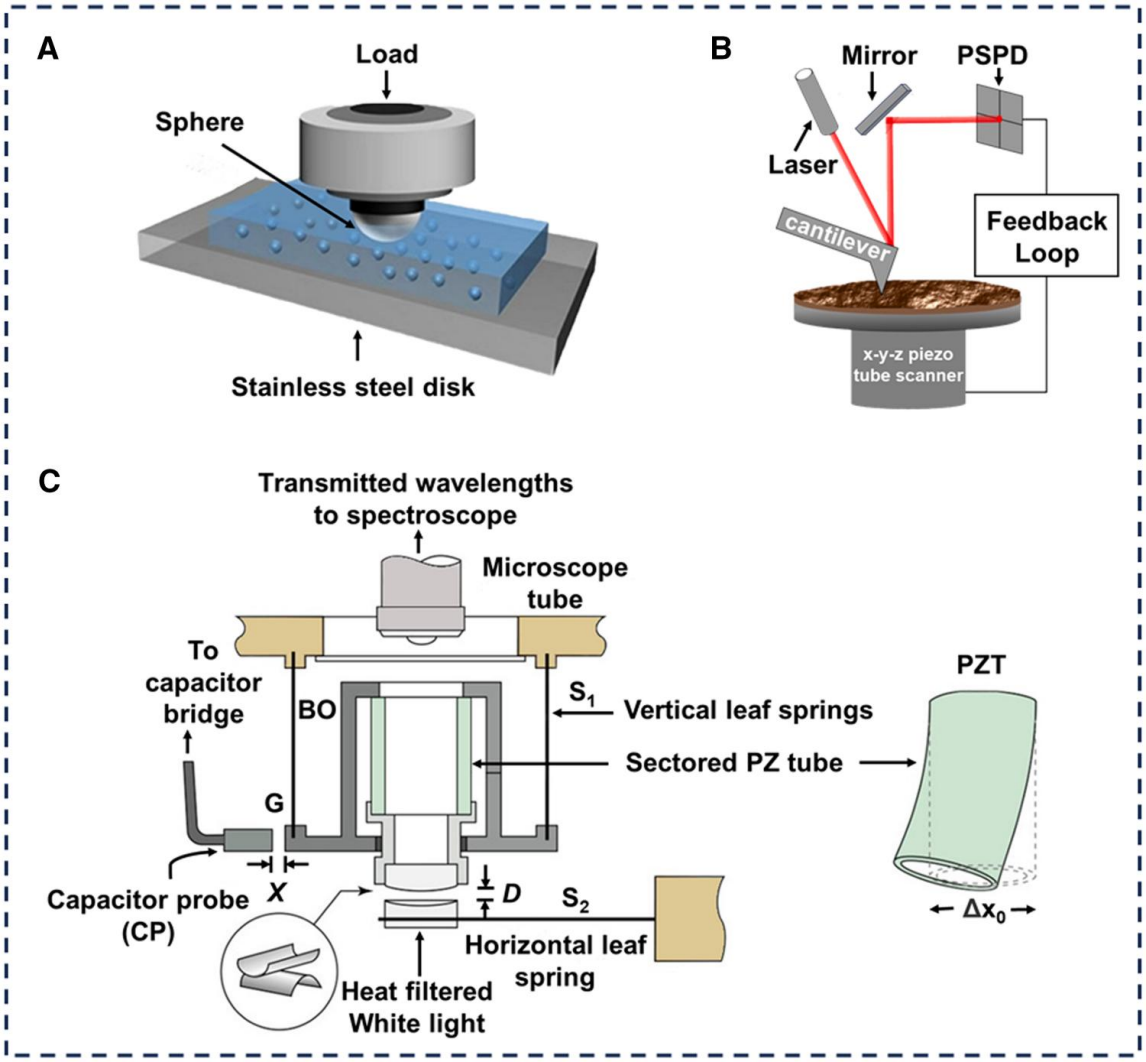
Three main instruments for measuring friction: (**A**) diagram of ball-on-disk tribometer. (**B**) Diagram of conventional scanning of AFM. (**C**) A schematic of the SFB which is used to measure normal and shear forces between molecularly smooth mica surfaces directly. (**A**) Reproduced with permission [55]. Copyright 2024, Elsevier. (**B**) Reproduced with permission [56]. Copyright 2022, Elsevier. (**C**) Reproduced with permission [29]. Copyright 2021, Wiley.

To evaluate the efficacy of ocular bio-lubricants and lubrication models, various friction testing instruments are required to simulate the complex interactions in ocular environments. These instruments vary in their validity for bio-lubrication testing, with each suited to specific aspects of the tribological behavior of biological or bio-inspired lubricants. Three prominent testing platforms—tribometers, AFM and SFBs—are widely employed (as shown in Figure 4). These tools enable critical evaluations of friction measurements, lubricant film thickness assessment, surface interaction analysis, lubricant performance evaluation, and multi-scale lubrication characterization in biological systems (Table 1). By integrating these methodologies, researchers achieve comprehensive insights into lubrication phenomena from molecular-scale to macro-scale, thereby advancing mechanistic understanding and facilitating the optimization of lubricant formulations. The following sections detail the working principles, application scopes, and ophthalmic relevance of these technologies.

**Table 1.** Summary of measured COF with different systems in aqueous media

Tools	Tribo-pairs	Media	Conditions	COF	Ref.
Tribometers	Contact lens vs. quartz glass	PBS or the preservative-free contact lens care solution^a^	*P* > 380 Pa *v*_s_ < 32.0 mm/s	≈0.004	[46]
	Glass plate vs. soft contact lens and rabbit corneas	The tear-like fluid contains a mixture of proteins, mucin, and added lipids	*P* ∼ 10.7–26.7 kPa *v*_s_ ∼ 0.100 mm/s	0.006–0.015	[47]
	Borosilicate glass sphere vs. hydrogel	/	*P* ∼ 30.0–50.0 kPa *v*_s_ ∼ 63.0–6280 μm/s	≈0.025–0.075	[48]
AFM	Silica sphere vs. silica substrate	HEPES buffer solution^b^	*P* ∼ 16.5 ± 8.80 kPa *v*_s_ ∼ 6.10 μm/s	/	[49]
	Silica probe vs. silica wafer	Water	*P* < 16.9 MPa *v*_s_ ∼ 2500 nm/s	0.007	[50]
	Borosilicate glass probe vs. contact lens	PBS	*P* ∼ 6.00–30.0 kPa *v*_s_ ∼ 5.00–200 μm/s	0.020	[51]
SFB	Mica vs. mica	rhPRG4 and HA^c^	*P* ∼ 3.60 MPa *v*_s_ ∼ 0.140 μm/s	<0.005	[52]
	Mica vs. amorphous fluoropolymer-coated mica	Water or physiological-level salt solution	*P* ∼ 5.07 MPa *v*_s_ ∼ 5–9 nm/s	0.003–0.009	[53]
	Mica vs. mica	Dextran and stroma	*P* ∼ 32.0 kPa *v*_s_ ∼ none	/	[54]

a PBS: phosphate-buffered saline.

b HEPES buffer solution: 4-[2-hydroxyethyl]piperazine-1-[2-ethanesulfonic acid].

c rhPRG4: recombinant human PRG4.

### Tribometer

Tribometers are crucial instruments for measuring the tribological properties of materials, including COF and the wear-resistant macroscopic level [57]. They are vital for understanding surface interactions in fields such as engineering, materials science and mechanical industries. Common types of tribometers include pin-on-disk, ball-on-disk, four-ball, reciprocating tribometers and UMTs. By using a ball-on-disk friction system (polyacrylamide ball-glass plate) (as shown in Figure 4A), Wang *et al.* [6] evaluated the influence of pressure and sliding velocity on the tribological performance of ocular slow-release lubricants. Under simulated ocular conditions (sliding speeds of 1–100 mm/s and pressures of 10–22 kPa), they measured an ultralow COF (≈0.005), demonstrating the capacity of the lubricant for stable slow-release, hydration retention and exceptional tribological behavior. Subsequent work by the team replaced the friction pair of polyacrylamide ball-glass plates with polyacrylamide ball-glycerol ethoxylate/polyvinylpyrrolidone composite hydrogels, and the photo-crosslinked composite hydrogel was found to have a super-lubricating effect (COF ≈ 0.005) under the same test conditions [58].

The UMT encompasses all modes and functions of the previously mentioned instruments. As a result, it is widely used to characterize friction processes and properties in fields such as industrial machinery, equipment, and biomedicine. Using a conventional UMT test model, Fang *et al.* [46] investigated the lubrication performance of contact lenses. In their experimental setup, a contact lens was mounted within a polyethylene (PE) specimen holder as the upper stage, while a quartz glass was used as the lower stage. PBS or preservative-free contact lens care solution was introduced to the lower stage. After applying a predetermined normal load by lowering the upper stage, rotational motion was initiated in the lower stage to measure frictional forces. Although the study evaluated lysozyme adsorption and the lubrication properties of preservative-free contact lens care solutions, the simplified model inadequately replicated ocular lubrication conditions, limiting its translational relevance to *in vivo* ocular environments. To address these limitations, Angeles *et al.* [59] improved the ocular model by replacing the original spherical upper component with a silicone rubber hemispherical fixture (mimicking corneal curvature) integrated with a contact lens, while substituting the quartz glass lower stage with a vulcanized silicone plate. The silicone rubber was selected for its biomedical congruence with natural cornea tissue, specifically its Young’s modulus. The silicone plate served as an eyelid model to more accurately simulate ocular conditions and assess the frictional characteristics of contact lenses.

While tribometers demonstrate configurational adaptability, their suboptimal force resolution and dynamic process monitoring deficiencies are significant constraints on the comprehensive characterization of ocular lubrication dynamics. This inherent constraint has driven the adoption of advanced nanomechanical characterization platforms—notably AFM and SFB—which provide angstrom-level resolution for real-time quantification of bio-lubrication energetics and molecular-scale shear dissipation mechanisms.

### Atomic force microscopy

AFM is a novel technique that uses a sharp tip to scan a sample with demonstrated resolution on the order of nanometer (as shown in Figure 4B) [60]. This scanning probe technique enables simultaneous topographical mapping and quantitative nanomechanical profiling through multifunctional cantilever dynamics including precise COF determination with nN to μN-scale force sensitivity [61]. AFM operates in three modes: contact mode, non-contact mode and tapping mode, each suitable for different types of sample surfaces. Lateral force microscopy (LFM), also known as friction force microscopy, is a branch of AFM contact mode. In LFM measurements, the photodiode quadrant detector tracks cantilever torsion via laser beam deflection, converting lateral displacement into frictional force through Hookean spring mechanics [62]. LFM has several advantages: a tiny contact area allows localized friction measurements, the surface and actual contact areas are nearly identical, and purely elastic conditions can be achieved with sufficiently low loading forces [63]. Additionally, it can simulate interactions at individual surface contacts under high contact pressures, making it particularly useful for studying friction under boundary lubrication conditions [64]. Shi *et al.* [65] utilized AFM to investigate the tribological and antifouling properties of silicone hydrogel (SiHy) contact lens materials made of 2-hydroxyethyl methacrylate and 2-methacryloyloxyethylphosphatidylcholine (MPC) copolymers. The advantage of LFM is its dual capability to quantify nanoscale friction forces while simultaneously imaging the morphology of the hydrated MPC polymer layer on the lens surface with sub-nanometer resolution, thereby facilitating the analysis of the correlation between frictional behavior and the structural organization of the hydrophilic surface domains. At the same time, the interaction forces between the AFM probe and lens surface were analyzed as a function of tip-sample separation distance. These forces exhibit a direct proportionality to the density of adsorbed proteins on the material surface, evaluating the anti-protein adsorption performance of MPC-modified silicone hydrogels—a critical determinant of biocompatibility in ocular applications. When conducting force profile measurements using conventional AFM, several critical issues arise if traditional probes are used directly: (i) the probe tip has an irregular geometry, making it challenging to establish a geometric model of the interaction between the probe and the sample. (ii) The probe tip is tiny (about 1–10 nm), resulting in a nanoscale contact area between the sharp tip and the sample surface, leading to poor measurement sensitivity and accuracy, especially for soft materials. (iii) Conventional tips are predominantly silicon/silicon nitride, restricting simulations of diverse material interactions (e.g. bio-lubricated interfaces) critical for engineering or biological applications. To overcome these limitations, colloidal probe AFM has emerged as a transformative solution. This method involves attaching micron- or nanoscale colloidal particles to the probe tip. The colloidal particles, typically ranging from several hundred nanometers to tens of micrometers in diameter, are fabricated from materials such as silicon, glass, polymers, or even biological cells [66]. Claesson *et al.* [67] employed colloid probe AFM to investigate interfacial interactions and frictional behavior between poly(methyl methacrylate) surfaces functionalized with distinct mucin coatings. During approach-retraction cycles, force-distance profiles revealed steric repulsion dominated by hydrated mucin layers upon approach, followed by minimal adhesion (<1 nN) during separation, indicative of weak interfacial cohesion. Frictional forces were quantified under incrementally applied normal loads (0–50 nN), enabling precise evaluation of mucin lubrication efficiency. The spherical geometry of the colloidal probe (5-µm silica particle) and enhanced contact area proved pivotal in resolving subtle differences in adsorption kinetics and load-dependent friction between mucin variants. Notably, mucin A exhibited a 40% reduction in COF (µ ≈ 0.02) compared to mucin B (µ ≈ 0.035), correlating with its superior surface coverage and hydration retention. The extremely high spatial resolution of AFM enables it to analyze the microscopic distribution of tear film components on the corneal surface and interfacial interactions, which is difficult to achieve using macro- or mesoscale friction techniques such as UMT and SFB. However, AFM measurements involve extremely small contact forces and contact areas, which may not fully simulate the macroscopic friction behavior between the eyelid and eyeball under physiological conditions. Additionally, unlike SFB, AFM cannot directly measure molecular-level membrane thickness or lubricant-limited membrane behavior, resulting in certain scale differences in terms of physiological relevance.

### Surface force balance

The SFB, also known as the surface force apparatus (Figure 4C), operates on a distinct principle compared to AFM. The SFB achieves sub-ångström resolution (1–2 Å) in real-time measurements of the absolute separation between two surfaces, whether in ambient air or immersed in nanoconfined liquids. This is accomplished via multibeam interferometry, which tracks shifts in the wavelengths of isochromatic fringes to determine surface separation. By applying Hooke’s law to measure the deflection of vertically and horizontally aligned springs, the SFB quantifies both normal and shear forces between surfaces, enabling direct calculation of COF. Klein [68] later optimized and enhanced SFB, improving its sensitivity and resolution, while expanding its utility to study interactions between surface-tethered polymers. These advancements addressed critical limitations of scanning probe and colloidal force microscopy, which only measure relative surface separations, thereby advancing the mechanistic understanding of bio-lubrication systems with unparalleled accuracy [69]. Pham *et al.* [70] used SFB to investigate the efficacy of BB polymers combined with HA in lubricating cornea and contact lens surfaces *in vitro*. Specifically, the three-block BB polymer combined with HA reduced the COF of dry cornea *in vitro* by 50%. While UMT focuses on macroscopic friction behavior in engineering applications, SFB and AFM specialize in microscopic interfacial analysis. More specifically, AFM excels at mapping nanoscale topography and local forces with ultra-high spatial resolution, while SFB enables quantitative measurement of interaction forces and lubrication properties in confined films under precise control of separation distance and alignment. SFB uniquely bridges molecular-scale interactions with macroscopic tribological performance, making it indispensable for both fundamental research and applied biomaterial innovation.

## Bio-lubricants for DES

To counteract pathological friction-induced damage, the human body employs a complex, multi-component biological lubrication system. While these systems operate across diverse physiological environments—subject to variable mechanical loads, pH levels, and biological fluid compositions, they universally achieve adequate lubrication under native physiological conditions. This functional robustness arises from synergistic multiple lubrication mechanisms (e.g. boundary, hydrodynamic, and hydration lubrication). Disruption of these natural lubrication mechanisms necessitates external interventions and treatments. DES, a prevalent ocular disorder affecting 5–50% of the global population depending on demographics, exemplifies lubrication failure in low-load, open-organ systems such as the ocular surface [20, 71]. Characterized by tear film instability and elevated interfacial friction, DES remains challenging to fully resolve, underscoring the need for bio-inspired lubricant design. Understanding bio-lubricants that can treat DES provides valuable insights for the development and application of lubrication materials for human bodies. Table 2 presents a list of common DES treatment methods, which are discussed in more detail later.

**Table 2.** Therapeutic strategies for DES and changes in COF before and after treatment

Treatment methods	Conditions	Tools	Initial COF	COF after treatment	Ref.
Artificial tears	*P* > 1.00 Pa *v*_s_ ∼ 3.00 μm/s	SFB	0.130	0.025	[70]
	*P* ∼ 10.0–22.0 kPa *v*_s_ ∼ 1.00–100 mm/s	Tribometer	0.200	0.040	[6]
	*P* ∼ 3.18 MPa *v*_s_ ∼ 100 μm/s	Tribometer	0.505	0.032	[72]
Contact lens	*P* ∼ 10.0–21.0 kPa *v*_s_ ∼ 1 mm/s	Tribometer	≈ 1.040	≈ 0.500	[73]
	*P* < 80.0 kPa *v*_s_ ∼ 0.100 mm/s	Tribometer	≈ 1.000	≈ 0.010-0.100	[18]
	*P* ∼ 5.00–9.00 kPa *v*_s_ ∼ 1–100 mm/s	Tribometer	0.014	0.005	[58]
	*P* ∼ 8.00–9.00 MPa *v*_s_ ∼ 4.00 μm/s	AFM	1.200	0.240	[67]
Drug vehicle	*P* ∼ 4–20 kPa *v*_s_ ∼ 0.3 mm/s	Tribometer	0.080	0.040	[74]

### Artificial tears

**Figure 5.**
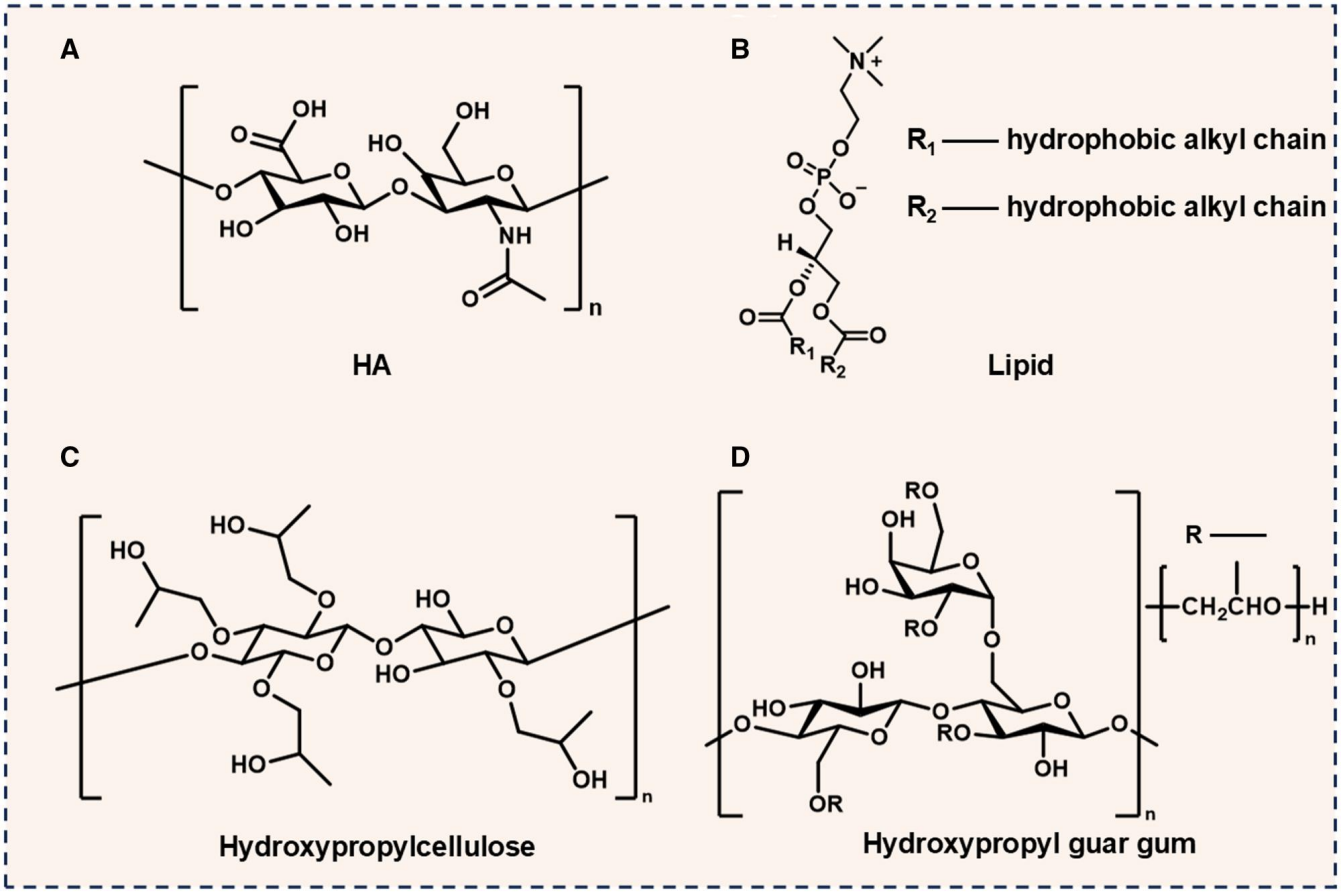
Chemical structure of lubricating molecules: (**A**) hyaluronic acid (HA), (**B**) lipid, (**C**) hydroxypropylcellulose (HPC) and (**D**) hydroxypropyl-guar gum (HPGG).

Artificial tears constitute the first-line therapeutic intervention for DES, functioning as bioengineered substitutes for endogenous tear secretion. Contemporary formulations incorporate lubricating molecules such as HA, lipids, cellulose derivatives and hydroxypropyl-guar gum (HPGG) (as shown in Figure 5) to achieve multimodal therapeutic effects [75, 76]. The main purpose of artificial tears is to replenish natural tears, reduce evaporation, enhance corneal hydration and lubricate. Furthermore, these formulations serve to preserve ocular surface integrity by sustaining glycocalyx function and modulating inflammatory cascades associated with chronic dry eye pathogenesis [75].

HA is a linear, anionic, non-sulfated glycosaminoglycan and demonstrates unique structural duality critical for ocular applications [77]. Unlike typical hydrophilic polymers, the axial hydrogen atoms (C–H) in the structure of HA form extensive domains of hydrophobic membrane sheet structure, where hydrophobic patches and highly hydrophilic groups coexist in the main chain of HA. This amphiphilic structure enables HA to simultaneously interact with aqueous tear components and hydrophobic ocular surfaces, establishing its superiority as a bio-lubricant in artificial tear formulations [78]. HA naturally occurs in a variety of tissues of the human body and serves critical biophysical and homeostatic functions in ocular surface physiology [79, 80]. However, native HA exhibits suboptimal ocular pharmacokinetics [80, 81]. Lee *et al.* [74] developed a polymer-peptide system with significantly prolonged HA retention on the ocular surface as well as kept it lubricated. This approach reduces the frequency of HA application and enhances therapeutic efficacy. Additionally, HA can be chemically modified to improve its rapid *in vivo* degradation by hyaluronidase. Through rational molecular design, Burgalassi *et al.* [82] developed HA functionalized with a metalloproteinase inhibitor. The engineered conjugate exhibits enhanced enzymatic resistance to hyaluronidase-mediated degradation, mediated through dual MMP-inhibitory functionality, which highlights potential translational applications for optimizing DES therapeutics.

Lipid constituents in artificial tear formulations prevent tear film evaporation through biomimetic stabilization of ocular surface architecture to protect the ocular epithelium. The lipids used in commercial formulations are mineral oil, castor oil, phospholipids (such as PC and hydrogenated phospholipids), omega-3 fatty acids, and medium-chain triglycerides. Phospholipids are crucial in maintaining tear film homeostasis through two synergistic mechanisms: monolayer formation [83] and vesicle transport [84]. A representative formulation is Tears Again^®^ Eye Spray, whose primary constituents self-assemble into unilamellar vesicles featuring an aqueous core enveloped by a phospholipid bilayer. Upon blink-induced shear stress, this lamellar structure undergoes controlled phospholipid release, enabling the integration of stabilized liposomal assemblies into the tear film matrix through hydrophobic interactions, thereby enhancing tear film structural integrity via lamellar phase stabilization [85]. Based on a hydration lubrication mechanism, Yang *et al.* [86] developed a novel bifunctional lubricating copolymer. This copolymer integrates two functional monomers: MPC and acrylate-N-hydroxysuccinimide (AA-NHS), engineered to synergistically address ocular surface dysfunction. The MPC component employs an amphoteric architecture, featuring an amphoteric nature with anionic phosphate (–${\text{PO}}_{4}^{-}$) and cationic quaternary ammonium (–N^+^(CH_3_)_3_) groups. This amphoteric structure induces the formation of a tightly bound hydration shell [87]. This molecular design induces ion-dipole-mediated hydration, which results in a strong repulsive force between the two amphiphilic phospholipid bilayers, structuring a dense interfacial water layer and achieving ultralow COF. Concurrently, the AA-NHS moieties undergo covalent conjugation with corneal epithelial lysine residues through nucleophilic substitution. This bio-adhesive strategy enhances ocular surface retention by 6.8-fold compared to passive adsorption mechanisms. This material alleviates dry eye symptoms by reducing both friction and inflammation, demonstrating the potential of hydration lubrication as a therapeutic strategy for DES. Hoang *et al.* [88] synthesized catechol-functionalized polyamphoteric nanotherapeutics incorporating MPC, which demonstrated significant potential for the treatment of DES owing to their superior lubricating, antioxidant and anti-inflammatory properties, as well as their prolonged ocular residence time. The hydration lubrication from the MPC in the structure reduces the likelihood of polyamphoteric extruding under shear or compression, resulting in more excellent viscous dissipation and an 85.6% reduction in COF compared to the control group.

Cellulose derivatives are commonly used as a thickener for DES treatment [89]. For example, hydroxypropylcellulose employs a hydration-driven matrix expansion mechanism to achieve sustained tear film thickening, with measurable increases in lipid-aqueous interface thickness persisting for hours post-application [8]. HPGG is another polysaccharide derivative that can thicken the tear film layer through a network of hydrogen bonding [90] and retain high stability and viscosity consistency across a wide pH range [91]. Markoulli *et al.* [92] tested the efficacy of HPGG-containing emulsions for treating DES and found that the lipid layer thickness increased compared to saline controls. Additionally, HPGG effectively enhanced symptom relief of DES patients throughout short-term and long-term treatment periods.

Single-component artificial tears are less effective for severe DES patients due to limited mechanistic complexity. Emerging evidence supports the clinical superiority of multi-constituent lubricants that synergistically target diverse pathophysiological pathways. Vigo *et al.* [93] treated patients with DES of varying severity for 2 months using eye drops containing HA, trehalose and cationic liposomes composed of stearylamine and phospholipids. Clinical studies showed a significant therapeutic effect on experimentally damaged rabbit corneal epithelial cells compared to linear HA and trehalose alone. The significant improvement in dry eye symptoms was linked to the properties of crosslinked HA. HA forms a dynamic viscoelastic solution in water, providing mechanical protection to cells, retaining moisture, and lubricating resistant surfaces. The cationic liposomes can provide both non-polar and polar lipids to complement and increase the thickness of the lipid layer while improving the stability of its interface with the aqueous phase of the tear film. In addition, the positive charge in the structure of cationic liposomes contributes to the generation of electrostatic forces that ensure stable adsorption of tear film soluble proteins at the interface of the lipid layer. The synergistic effects of HA, trehalose, and cationic liposomes work together to induce a decrease in ocular tear evaporation and amelioration of DES. Artificial tears incorporate thickeners that reduce water evaporation and enhance drug retention by modifying tear film viscosity [94]. The viscoelastic properties of ophthalmic gels can be precisely modulated by adjusting polymer concentration gradients, enabling customization for varying clinical requirements. This rheological adaptability renders such formulations particularly advantageous for managing severe DES.

While short-term artificial tears application effectively alleviates DES symptoms, long-term application may introduce risks. A subset of patients develops allergies to ingredients, particularly preservatives, causing symptoms like redness, swelling, itching, or stinging. Long-term application of artificial tears can inhibit lacrimal gland function, reducing basal tear secretion and increasing dryness.

### Contact lens-based lubrication strategies

Wearing contact lenses may increase the risk of dry eye by reducing oxygen supply to the cornea, disrupting tear film stability, and accelerating tear evaporation. These changes can lead to ocular surface dryness and discomfort. Prolonged wear, inadequate lens care, or the use of low-oxygen-permeable lens materials may further contribute to inflammation and abnormal tear production. Common adjunctive treatments for contact lens-induced DES include comfort agents, lens immersion in multipurpose solutions, and modification of contact lenses. Among these, comfort agents emerge as the most rapid and effective intervention, functioning similarly to artificial tears by replenishing ocular lubricants to restore tear film integrity, improve ocular surface lubrication, and optimize contact lens comfort. As a bio-lubricant, HA is the most effective polysaccharide comfort agent. Fernández-Jimenez *et al.* [41] evaluated the efficacy of a bioprotective preservative-free, hypotonic, 0.15% HA-3% trehalose artificial tear in controlling dry eye symptoms in contact lens wearers. The combination of trehalose and HA can be used to lubricate the ocular surface through prolonged hydration for maximum comfort. After use, ocular inflammation and tear evaporation rates were significantly reduced, and adequate lubrication reduced discomfort from contact lens wear. An alternative treatment involves soaking contact lenses in a multipurpose solution that continuously releases ocular lubricant during use. HPMC is an effective lens moisturizer, improving tear film function and stabilizing its structure. Thai *et al.* [95] employing both *in vivo* and *in vitro* models revealed that HPMC-enriched solutions create a durable, thickened liquid interface on lens surfaces through continuous lubricant release during wear.

**Figure 6.**
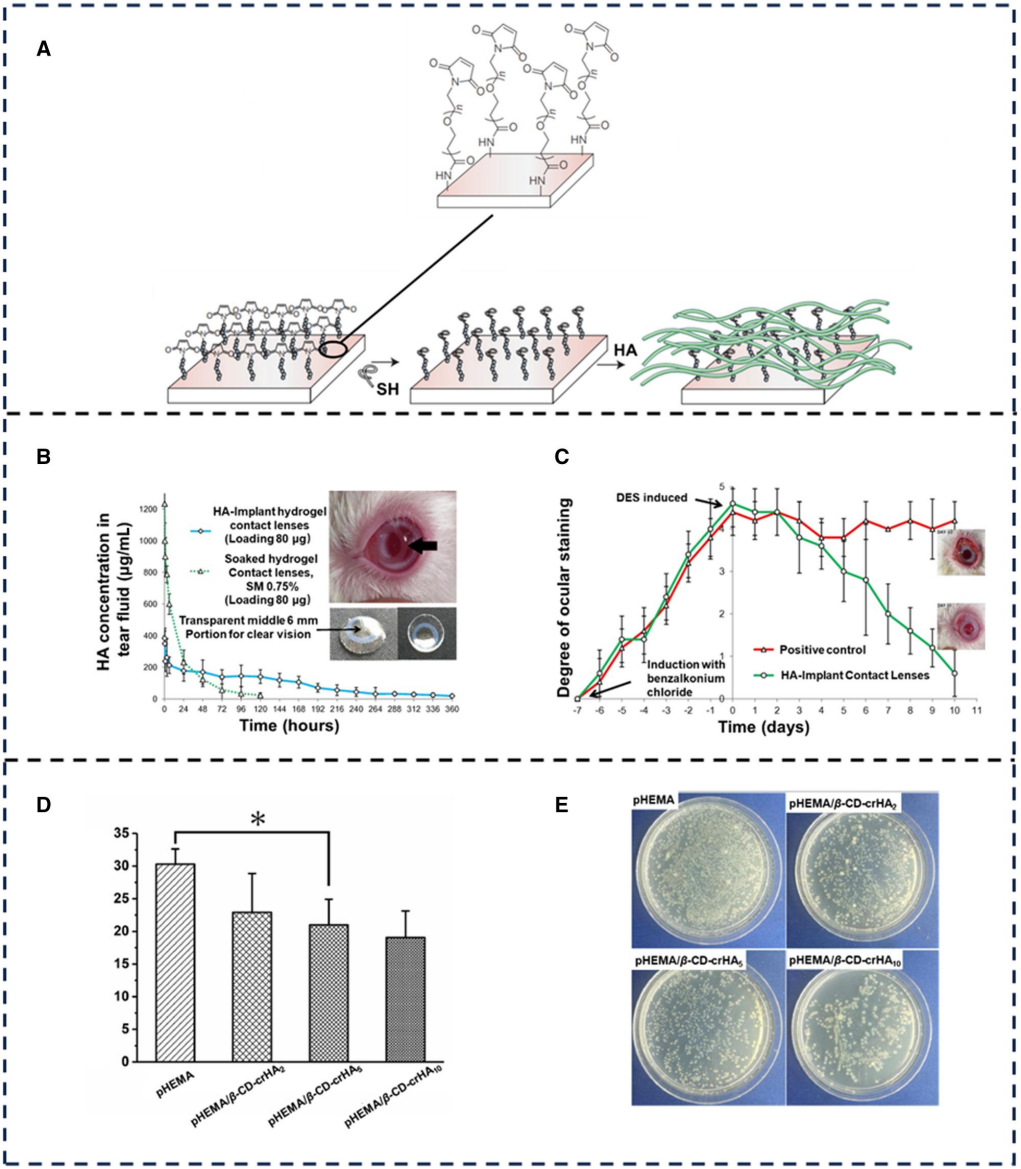
Contact lenses that can be used for ocular lubrication. (**A**) The HABpep-polymer system as an eye-drop solution can be used to retain HA on the eye surface. Contact lens modification with the HABpep-polymer system was performed by the covalent reaction methodology. (**B**) HA tear fluid concentration-time profiles after application of sterilized HA implant contact lenses (loading 80 μg) and (**C**) soaked contact lenses (SM 0.75%, loading 81.84 μg). Values are shown as mean ± SD (*n* = 6). (**D**) Total protein adsorption of various hydrogels in a simulated tear solution at 37°C for 12 hours, and (**E**) adhesion of *Staphylococcus aureus* on the surface of above protein-adsorbed hydrogels evaluated by a colony growth method. Error bars represent mean ± SD (*n* = 3). (*P < 0.05). (**A**) Reproduced with permission [99]. Copyright 2023, Elsevier. (**B**) and (**C**) Reproduced with permission [97]. Copyright 2017, Elsevier. (**D**) and (**E**) Reproduced with permission [98]. Copyright 2020, Elsevier.

While the methods described above can provide transient restoration of ocular physiology, their long-term efficacy is constrained by dependency on repetitive comfort agent administration, which risks blurred vision and transient visual disturbances due to overdosing. This limitation has driven innovation in bio-functional lens modifications that integrate lubricating molecules directly into contact lens materials, enabling sustained therapeutic action. Singh *et al.* [96] pioneered a biomimetic approach by applying HA bound to HABpep-polymer as an eye drop with the polymer-peptide system anchored onto type I collagen of the sclera, conjunctiva and cornea of an eye through collagen I binding peptide. The contact lenses with HA adhesive were evaluated for their effect on the water evaporation rate, showing a significant reduction in water loss (as shown in Figure 6A). Maulvi *et al.* [97] implanted an HA loading ring into hydrogel contact lenses, enabling continuous diffusion of HA into the lenses and tear film (as shown in Figure 6B and C). The HA-loaded contact lenses were well-modified and sustained HA release within the therapeutic range for up to 15 days. The HA-treated contact lenses demonstrated significant improvement in comfort. Li *et al.* [98] crosslinked β-cyclodextrin and HA with poly(methyl methacrylate) (2-hydroxyethyl) ester to develop a tear-protein adsorbent-resistant material for SCLs with enhanced wettability (as shown in Figure 6D and E). Functional contact lenses produced using these methods can keep the eyes moist for extended periods. However, their friction behavior during use remains uninvestigated.

**Figure 7.**
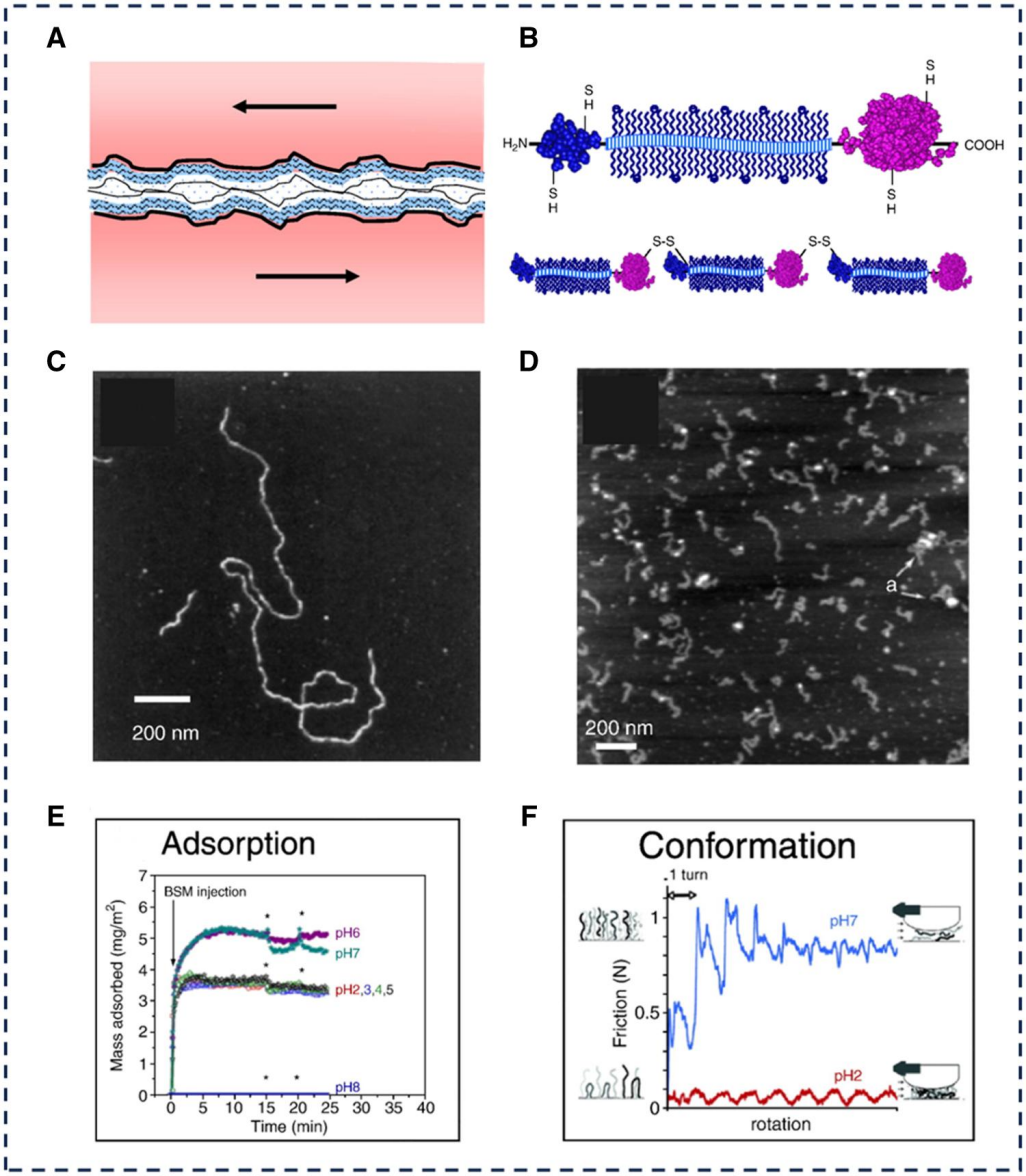
The structure and lubrication process of mucin. (**A**) The lubrication process of mucin. (**B**) A typical mucin subunit showing a central linear glycosylated domain flanked by globular domains (above) and a mucin molecule composed of subunits linked linearly by disulfide bonds (below). AFM high-resolution images showing (**C**) isolated ocular mucin and (**D**) PRG4. Lubrication by mucins is complex and depends, among other things, on (**E**) the extent of mucin adsorption and (**F**) surface conformation and structure of the adsorbed mucin. Reproduced with permission [103]. Copyright 2010, Elsevier.

Mucins are a diverse family of proteoglycans found widely in soft lubricating interfaces, existing as membrane-bound and gel-forming substances. Mucins contain both hydrophilic (glycosylated) and hydrophobic (non-glycosylated) domains, making them amphiphilic block copolymers capable of adhering firmly to various surfaces via hydrogen bonding, hydrophobic interactions, and electrostatic forces (as shown in Figure 7) [39]. As a result, mucins adhere well to ocular epithelial cells, forming a bio-lubricating coating that can modify contact lens surfaces [100]. Carolin *et al.* [101] applied a visco-protein macromolecule coating to the surface of contact lenses via covalent coupling. The results showed that coating the lenses with mucin significantly reduced the COF of the lens surface. Negatively charged mucins are believed to play roles in hydration, protection, and lubrication of the ocular surface, while also retaining antimicrobial proteins and resisting the adhesion of particles and pathogens [102]. Proteoglycan 4 (PRG4), also known as lubricin, is a protein found within mucins, usually as a monomer or disulfide-bonded dimer. Like mucins, PRG4 has smaller globular amino- and carboxy-terminal regions, separated by a large, highly glycosylated central domain. While mucins are generally negatively charged, PRG4 is slightly positively charged at physiological pH due to the abundance of lysine and arginine residues in its protein core [103]. It adsorbs to the epithelial cell surface via hydrophobic interactions between its C-terminal domain and the eye surface, forming a low-friction, polymeric brush-like lubricant layer of negatively charged glycan chains. This layer remains stable even in thin liquid films, making it an important ocular surface boundary lubricant [104]. Rotational rheometer experiments have shown that PRG4 is an indispensable lubricating molecule in living organisms [105]. The interaction and synergistic lubrication of PRG4 with other molecules in the tear film through non-covalent entanglement contribute to ocular super-lubrication. PRG4 is ideal for reducing ocular friction on contact lens surfaces and may maintain a low COF even when the tear film is damaged. In 2015, Samsom *et al.* [106] conducted an *in vitro* friction test on SiHy with attached PRG4. The adsorption of PRG4 on the lens surface formed a boundary lubrication layer, greatly reducing surface friction and exemplifying surface modification of CLs. Korogiannaki *et al.* [107] chemically grafted full-length recombinant human PRG4 onto the surface of SiHy contact lenses via its somatomedin B-like terminal domain using *N, N′*-carbonyldiimidazole. The surface was then impregnated with poly(2-hydroxyethyl methacrylate), enhancing water retention and lubricity. The surface contact angle and COF of PRG4 were reduced compared to the ungrafted lens surface, confirming results similar to those of Samsom. In summary, PRG4 is a superior lubricant that protects the human cornea from shear stress caused by the eyelid and other biological materials.

**Figure 8.**
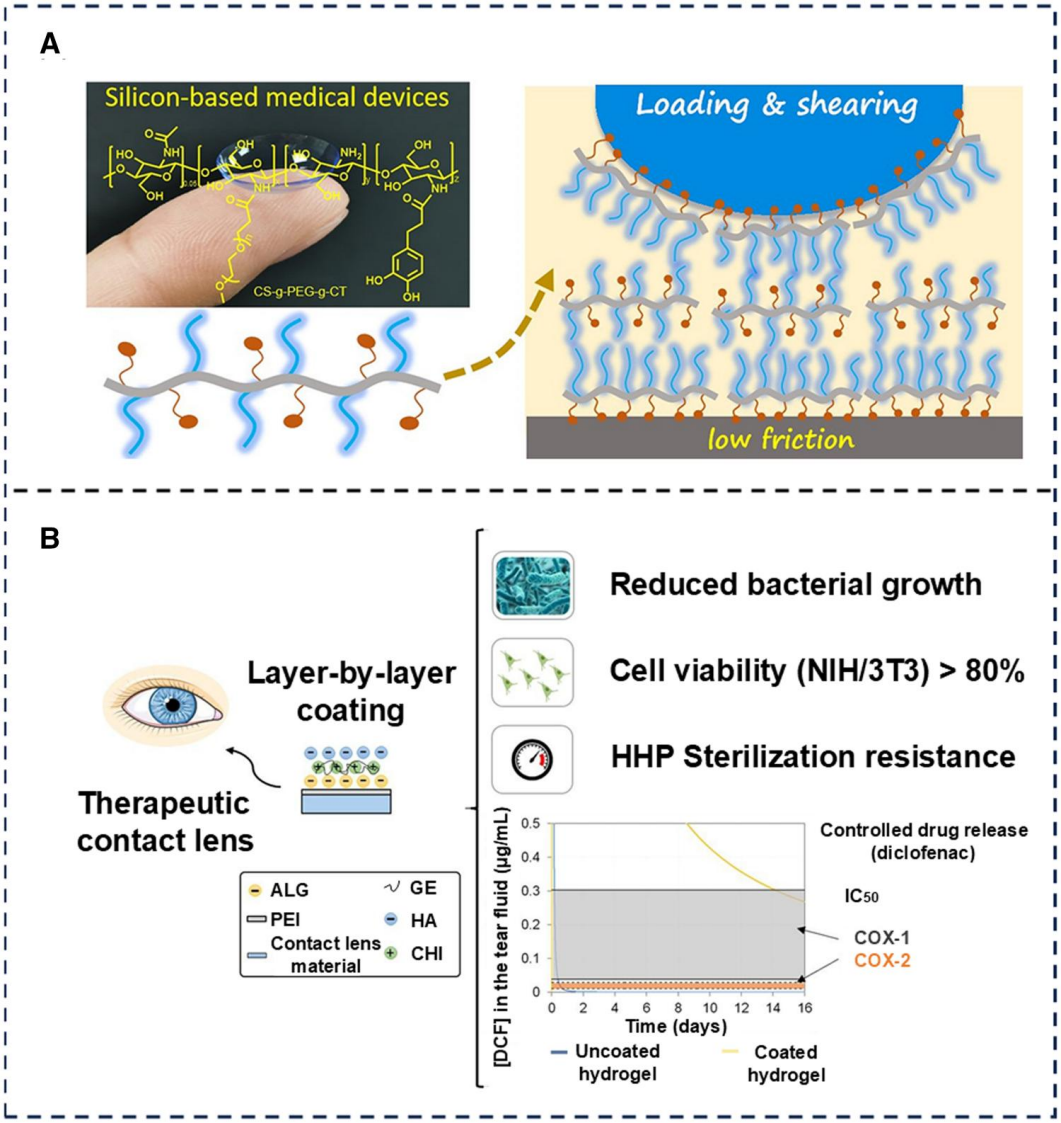
Chitosan plays an important role in wound healing and contact lens modification due to its favorable biocompatibility and antimicrobial activity. (**A**) The lubrication mechanism of CS-*g*-PEG-*g*-CT on silicon substrates. (**B**) A layer-by-layer coating was designed using ionic polysaccharides (chitosan, sodium alginate, sodium hyaluronate) and genipin (crosslinker), to sustain the release of diclofenac sodium salt (DCF) from soft contact lens (SCL) materials. The coating was hydrophilic, biocompatible, non-toxic and reduced bacterial growth. (**A**) Reproduced with permission [109]. Copyright 2022, American Chemical Society. (**B**) Reproduced with permission [111]. Copyright 2020, Elsevier.

A variety of lubrication modifiers for hydrogel contact lenses exist, most of which are biomolecules naturally found in the human body. Chitosan, found in the shells of crustaceans and certain fungi, is frequently utilized as a boundary lubricant due to its amine group, which enhances hydrated ion adsorption and hydration lubrication [108]. This property holds the potential for alleviating dry eye symptoms caused by contact lens wear. Gao *et al.* [109] developed a novel macromolecular bio-lubricant (chitosan-*g*-PEG-*g*-CT) composed of chitosan-grafted polyethylene glycol (PEG) chains and catechol moieties (CT) (as shown in Figure 8A). Chitosan-*g*-PEG-*g*-CT adsorbs onto poly(dimethylsiloxane) surfaces, demonstrating excellent lubricating properties (COFs 0.01–0.02) under physiological conditions. With the increasing development and application of bio-lubricants, contact lens manufacturing materials are no longer restricted to single polymer hydrogels. Alginate, a polysaccharide derived from seaweed, crosslinks with multivalent cationic calcium ions in the eye to form a natural macromolecular hydrogel, paving the way for the development of naturally lubricated contact lenses [110]. Silva *et al.* [111] developed a layer-by-layer coated SCL by integrating the properties of three ionic polysaccharides: alginate, chitosan, and HA (as shown in Figure 8B). This SCL exhibits favorable hydrophilic and antimicrobial properties; however, tribological tests were not conducted, and it remains uncertain whether the alginate hydrogel can provide both antimicrobial and lubricating effects, as well as comfort for the wearer.

### Novel lubricating materials with drug delivery

Recent advancements in nanotechnology have introduced new strategies to overcome the limitations of traditional drug delivery systems, with the core focus on developing nanostructures capable of efficiently encapsulating and precisely delivering small-molecule drugs. In ophthalmic therapy, nanocarriers demonstrate multiple advantages, including significantly enhancing the bioavailability of local drug administration, enabling controlled drug release and targeted delivery, thereby effectively improving overall treatment outcomes. Studies have shown that drug-loaded nanoscale systems used for the treatment of anterior segment eye diseases, commonly referred to as “nanodrugs”, feature low dosage requirements, prolonged retention times on the ocular surface, and reduced administration frequencies. These characteristics collectively contribute to a significant improvement in patient tolerance and medication adherence [112]. For the treatment of DES, nanocarriers not only alleviate symptoms but also intervene in its pathological mechanisms, successfully overcoming the shortcomings of traditional artificial tears, such as short duration of action and low bioavailability. Common nanomedicine carriers and ocular surface lubrication systems include lipid nanoparticles, nanoemulsions, dendrimers, polymer nanoparticles, and other novel drug delivery systems. These systems, with their high affinity for ocular surface tissues, can significantly prolong drug retention time in front of the cornea and enhance their penetration through the ocular biological barrier, thereby markedly improving ocular bioavailability [113].

As the earliest lipid nanoparticles, liposomes have good corneal permeability, high drug-carrying efficiency, and sustained release kinetics due to their structural resemblance to biological membranes, allowing them to successfully move from conceptual to clinically applicable nanodrug delivery platforms. Ren *et al.* [114] designed a novel ocular delivery vehicle containing liposomes of azithromycin (AZM) for the treatment of DES. It is highly efficient and superior compared to AZM solutions, not only for the use of the liposomal carrier for ocular lubrication but also for improving the compatibility of AZM with the lipid layer in the tear film structure, increasing corneal permeability by a factor of 2-fold. However, conventional liposomes require complex production methods using organic solvents, are inefficient at encapsulating drugs, and are difficult to perform on a large scale. Nanostructured lipid carrier (NLC) consists of a mixture of solid and liquid crystalline lipids that exhibit enhanced physical stability, addressing one of the major limitations of liposome-based carriers. Tan *et al.* [115] developed NLCs loaded with dexamethasone (DEX) and (3-aminomethylphenyl)boronic acid conjugated with chondroitin sulfate (APBA-CS). The DEX-APBA-CS-NLC carrier utilizes lipids to provide lubrication for the eyes, while the boric acid molecules on the surface form complexes with ocular mucus, further prolonging drug retention time in the anterior segment of the cornea, with bioavailability 6.8 times that of DEX eye drops. This system effectively alleviated DES and provided a reference for subsequent studies. Lipid nanoparticles drug delivery systems represent an important direction in DES therapy, and their development is not only expected to improve patient outcomes and quality of life but also provide lessons for drug delivery in other ocular diseases. With the continuous advancement of nanotechnology and material science, ocular delivery systems will develop in the direction of being more efficient and safer.

At the same time, nanoemulsions, as another promising nanocarrier, have demonstrated unique advantages in the field of ocular drug delivery. Nanoemulsions, as colloidal dispersion systems composed of oil phase, water phase, surfactants and co-surfactants, demonstrate significant advantages in the field of ophthalmic drug delivery. Their particle size typically ranges from 10 to 1000 nm, exhibiting kinetic stability and optical transparency, making them suitable for ocular administration. Nanoemulsions can efficiently encapsulate lipophilic and partially hydrophilic drugs, significantly enhancing drug solubility and corneal permeability. The gatifloxacin microemulsion developed by Kalam *et al.* [116] not only exhibits excellent stability but also forms strong adhesion on the corneal surface, doubling the drug concentration in the anterior chamber compared to traditional formulations. This is attributed to its compatibility with the lipid layer of the tear film and the temporary reversible modulation of tight junctions between corneal epithelial cells by surfactants. Kassaee and Mahboobian [117] prepared a besifloxacin nanoemulsion that demonstrated good controlled-release properties and corneal permeability in the eye. *In vitro* experiments showed that its corneal permeability was 1.7 times higher than that of besifloxacin suspension, and it did not cause tissue damage in a bovine eye model, demonstrating good biocompatibility. Notably, despite the lower drug concentration (0.2%) of the nanoemulsion compared to the traditional suspension (0.6%), its therapeutic efficacy was comparable. However, the widespread application of nanoemulsions still faces challenges, including potential ocular irritation caused by high concentrations of surfactants and issues related to physical stability during long-term storage. To overcome these limitations, researchers are developing novel, safe and efficient emulsifier systems and further extending their retention time on the ocular surface through surface modification (such as chitosan coating), thereby providing new strategies for achieving efficient and safe ocular therapy.

In parallel to emulsion-based systems, dendrimers represent another structurally distinct and functionally versatile class of nanocarriers that have garnered considerable attention for ophthalmic applications. Dendrimers are a class of nanoscale polymers characterized by highly branched three-dimensional structures, typically featuring a tree-like or star-shaped topological morphology composed of repeating units radiating outward from a central core. The physical dimensions and spatial configuration of these macromolecules are determined by the number and arrangement of their core arms, while surface functional groups dictate their physicochemical properties and biological activity [118]. Thanks to their unique structure, dendrimers can efficiently encapsulate large amounts of hydrophilic or hydrophobic drugs and can further expand their functional diversity as nanocarriers through surface modification, demonstrating significant advantages in the treatment of ocular surface inflammatory diseases such as DES and in ocular drug delivery. Soiberman *et al.* [119] developed a dendritic polymer gel combining HA and DEX, which significantly reduced corneal thickness and inflammatory responses in a rat model after subconjunctival injection, while avoiding increased intraocular pressure. *In vitro*, the gel demonstrated excellent anti-inflammatory activity in lipopolysaccharide-activated macrophages (approximately 1.6 times more potent than free DEX at a concentration 10 times lower). This system can specifically target inflammatory cells, improving corneal transparency while minimizing the side effects of steroid drugs, highlighting the application potential of dendritic macromolecules as efficient and safe ocular drug delivery carriers.

Beyond the highly ordered architecture of dendrimers, polymer-based nanoparticles constitute another major category of nanocarriers that leverage versatile material properties and formulation designs for enhanced ocular drug delivery. Polymer nanoparticles are important carriers in ocular drug delivery and can be classified into two types: nanospheres (where the drug is uniformly dispersed within the matrix) and nanocapsules (where the drug is encapsulated within a polymer shell). Their particle size is typically <1000 nm, and they exhibit excellent mucosal adhesion properties. Particles can be administered via topical eye drops, penetrating to the posterior segment of the eye through pathways such as the cornea-anterior chamber-vitreous pathway or the conjunctiva-sclera-choroid pathway, effectively prolonging drug retention time on the ocular surface and enhancing bioavailability. Modi *et al.* [120] developed ion-sensitive gel-loaded tacrolimus nanoparticles (TGNP) based on carrageenan, which demonstrated sustained drug release and symptom relief in the treatment of DES. In *in vivo* studies, tacrolimus solution remained in the anterior corneal region for <2 hours, but TGNP demonstrated good corneal penetration, remaining in the anterior cornea for 12 hours. Although nanoparticle formulations can protect the drug and control release, there is currently a lack of efficient eye-drop formulations for delivering drugs to the retina, necessitating the development of new materials and optimized designs to advance clinical translation.

## Concluding remarks and perspectives

In conclusion, ocular super-lubrication is a highly regulated physiological phenomenon governed by the synergistic interactions of key biological lubricants—primarily PC, HA, and mucin-associated glycoproteins—within the tear film. These components contribute to the maintenance of a hydrated, low-friction interface essential for minimizing mechanical shear stress during blinking and ocular movements. Perturbations in the synthesis, secretion, or structural integrity of these lubricating agents disrupt tear film homeostasis, leading to increased frictional forces at the ocular surface. This mechanical damage compromises epithelial cell integrity, impairs regenerative processes, and initiates a chronic cycle of inflammation and tissue remodeling, which cumulatively contribute to the pathogenesis of DES. By synthesizing current knowledge on the biophysical mechanisms of ocular lubrication and the functional roles of endogenous lubricants, this review underscores the critical importance of preserving lubricant equilibrium and provides a rationale for the development of biomimetic therapeutic strategies targeting lubrication deficits in ocular surface disorders.

This paper provides an overview of key issues related to ocular lubrication, the study of which will contribute to the development of biological lubricants and expand our approach to designing and improving lubrication systems to address friction-related disorders. These issues include: (i) Two primary models for ocular lubrication have been developed: liquid film lubrication and boundary lubrication. However, the dynamics of blinking, including when and how these two models transition and which model predominates, remain to be investigated. (ii) The synthesis of natural lubricants in the eye is regulated by cellular genes, with a reciprocal interaction and feedback mechanism between lubricants, tissues and cells. The stability of the tear film directly influences ocular lubrication. (iii) A significant consequence of DES is the destruction of structural integrity, leading to irreversible damage. In the development of biological and biomimetic lubricant materials, there is an urgent need to overcome challenges in synthesizing biomimetic lubricants that exhibit the properties of natural tear gel-sol transformation and shear thinning, as well as improving the ocular compatibility of synthetic lubricants for sustained lubrication. The development of lubricant materials for DES is a current hotspot in chronic disease treatment. It is believed that exploring and understanding these issues can inform the treatment of ocular diseases, guide the further development of biomimetic lubricants and lubrication materials and promote the advancement of ocular tribology.

To address these challenges and drive progress in this field, it is essential to identify specific research directions and technical pathways.

1. Enhancing the biocompatibility and ocular surface adhesion of lubricating materials is of utmost importance. This can be achieved through surface modification strategies, such as developing biomimetic coatings inspired by the structure of natural mucoproteins, or using materials with excellent anti-protein adsorption and moisturizing properties, such as 2-methylacryloxyethyl PC. Additionally, advanced characterization techniques such as AFM and SFB can reveal nanoscale interactions between lubricants and the ocular surface, thereby guiding the design of materials with optimized biocompatibility.
2. Developing complex lubricant-drug co-delivery systems represents a promising research direction. Future research should focus on designing multifunctional platforms, such as supramolecular nanodrugs or injectable hydrogel microspheres, which can provide sustained lubrication while simultaneously releasing anti-inflammatory drugs (e.g. ketorolac or flurbiprofen). These systems should be capable of responding to specific pathological stimuli (such as pH, enzymes, or near-infrared light) to achieve on-demand drug release, thereby enhancing therapeutic efficacy and reducing side effects. Optimizing the synthesis of these systems to mimic the gel-to-solution transition and shear-thinning properties of the natural tear film is critical for clinical applications.
3. Translating these advanced materials and systems from the laboratory to clinical applications presents significant challenges. These challenges include scaling up production under good manufacturing practice conditions, establishing robust quality control metrics for complex biomimetic materials, designing predictive preclinical models that accurately replicate human ocular surface conditions, and conducting long-term safety and efficacy studies. Overcoming these obstacles requires interdisciplinary collaboration among materials scientists, bioengineers, pharmacologists, and clinical ophthalmologists to ensure that novel therapies are not only effective but also scalable, reproducible, and accessible to patients.
